# Sex-specific trends in hip fracture incidence and disparities in DXA utilization in Brazil: a nationwide ecological analysis

**DOI:** 10.1007/s11657-026-01734-5

**Published:** 2026-08-26

**Authors:** Isabela Januário, Ben-Hur Albergaria, Bruno Casaes Teixeira, Marise Lazaretti-Castro

**Affiliations:** 1 Universidade Federal de São Paulo, São Paulo, Brazil; 2 Universidade Federal Do Espírito Santo, Vitoria, Brazil; 3 Present Address: Independent Researcher, London, United Kingdom

**Keywords:** Hip fracture, Osteoporosis, Epidemiology, Bone densitometry

## Abstract

**Summary:**

This study analyzed sex-specific trends in hip fracture incidence, DXA utilization, and device distribution in Brazil from 2008 to 2022. Hip fracture incidence increased over time, whereas DXA use, particularly among men, remained markedly lower, with substantial disparities in device availability between the public and private sectors.

**Purpose:**

We aimed to evaluate temporal trends in hip fracture incidence and DXA exam utilization in Brazil, stratified by sex, and to assess DXA device distribution across public and private healthcare sectors.

**Methods:**

This was a retrospective analysis of Brazilian public healthcare databases from January 1, 2008, to December 31, 2022, examining hip fracture hospitalizations, DXA exam utilization, and DXA device availability.

**Results:**

From 2008 to 2022, 583,205 hip fractures occurred in individuals ≥ 50 years living in Brazil. In 2022, incidence rates were 155/100,000 in women and 94/100,000 in men; median ages were 80 and 74 years, respectively. Hip fracture incidence increased by 25.8%, with a more pronounced rise after 2015. DXA rates in 2022 were 2177/100,000 in women and 182/100,000 in men ≥ 50; in guideline-recommended groups, 2840/100,000 in women ≥ 65 and 385/100,000 in men ≥ 70. The hip fracture ratio between men and women was ~ 2:3, whereas the DXA exam ratio was 1:10. DXA device availability was 12.2 per million inhabitants overall, with 6.2 in the public sector versus 27 in the private sector.

**Conclusions:**

Hip fracture incidence in Brazil increased over the study period, particularly after 2015, without a proportional expansion in DXA utilization. This mismatch is more pronounced in men, who experience a substantial fracture burden but remain markedly underscreened. These findings highlight structural inequities in access to osteoporosis diagnosis and suggest persistent underdiagnosis, particularly in the public healthcare system.

**Supplementary Information:**

The online version contains supplementary material available at 10.1007/s11657-026-01734-5.

## Introduction

Osteoporosis is characterized by reduced bone mass and altered skeletal microarchitecture, which increases bone fragility and susceptibility to fractures [1]. Although commonly perceived as a disease predominantly affecting women, approximately one-third of all hip fractures worldwide occur in men [2, 3]. In Brazil, approximately 12.8% of men aged 40 years or older experience low-impact fractures, closely in line with the global estimate of 12% [4]. Men who sustain hip fractures have higher morbidity and mortality rates than women. However, osteoporosis remains frequently undiagnosed and undertreated in the male population [5–7]. Indeed, fewer than 20% of men receive osteoporosis treatment, including those with previous fracture history [2, 8]. These findings reveal persistent gaps in the evaluation of osteoporosis among men.

Current guidelines from specialized societies recommend at least one lifetime bone densitometry assessment via dual-energy X-ray absorptiometry (DXA), the gold standard for osteoporosis diagnosis, for all men aged 70 years and older and all women aged 65 years and older [9, 10]. However, evidence from international studies consistently demonstrates lower DXA utilization among men than among women, highlighting gender disparities in osteoporosis screening practices [11, 12]. Furthermore, regional and socioeconomic inequalities substantially influence access to osteoporosis screening, diagnosis, and preventive interventions, especially in regions where healthcare infrastructure and specialized resources, such as DXA scanners, are unevenly distributed [13].

In Brazil, the public Unified Health System (Sistema Único de Saúde, SUS) provides universal and free healthcare services to approximately 70% of the population, representing a fundamental pillar for healthcare accessibility [14, 15]. The remaining population relies primarily on private health insurance, although some individuals may still access public healthcare services. Within this context, the national health information system (DATASUS) provides publicly available administrative data from both hospital and outpatient care and has been increasingly used for epidemiological and health services research, offering an opportunity to evaluate real-world patterns of disease burden and healthcare utilization at a population level [16–21].

Previous estimates of hip fracture incidence in Brazil have been derived from both primary epidemiological studies with direct data collection and analyses of national administrative healthcare databases [19, 22–24]. The Brazilian Osteoporosis Study (BRAVOS) reported standardized annual incidence rates of approximately 130 per 100,000 women and 78 per 100,000 men aged ≥ 50 years, based on data collected between 2010 and 2012 [24]. More recent analyses using national administrative databases have described temporal trends in hip fracture incidence, reporting rates of approximately 116.9 per 100,000 in women and 71.2 per 100,000 in men between 2008 and 2018 [22]. However, updated nationwide analyses incorporating more recent data remain limited. In addition, studies describing DXA utilization patterns by sex and the distribution of DXA resources across healthcare sectors are scarce.

The aim of this ecological study was to describe age- and sex-specific trends in hip fracture incidence and DXA utilization in Brazil from 2008 to 2022 using national public healthcare databases, and to examine the distribution of DXA scanners across public (SUS) and private healthcare sectors.

## Methods

### Study design and data sources

This retrospective, observational, population-based study included data from 2008 to 2022. Population data for Brazil were obtained from the Brazilian Ministry of Health’s platform (TABNET/DATASUS), based on official demographic estimates of the total Brazilian population [25]. Data on private health insurance coverage were sourced from the National Supplementary Health Agency (ANS), which is responsible for regulating and supervising the private health insurance sector in Brazil [26].

Data on hospitalizations were obtained from the Hospital Information System of the Unified Health System (Sistema de Informações Hospitalares do SUS, SIH-SUS), and data on densitometry exams were retrieved from the Ambulatory Information System (Sistema de Informações Ambulatoriais do SUS, SIA-SUS), both available through the Department of Informatics of the Unified Health System (DATASUS), maintained by the Brazilian Ministry of Health [27]. Hip fracture and DXA utilization data, therefore, reflect healthcare services delivered within the Brazilian public healthcare system (SUS).

Device availability data were collected from the National Registry of Health Establishments (Cadastro Nacional de Estabelecimentos de Saúde, CNES), which includes both public and private healthcare facilities [28].

These administrative databases are widely used for epidemiological research and health system monitoring in Brazil [16–21]. All data sources are publicly accessible and anonymized; therefore, ethical approval was not required.

### Population denominator

Incidence and utilization rates were calculated using the estimated population covered by the public healthcare system. This denominator was defined as the total Brazilian population minus the population covered by private health insurance. This approach assumes that individuals covered by private health insurance are less likely to utilize public healthcare services.

### Hip fracture identification and incidence

Hip fractures were identified using International Classification of Diseases, tenth revision (ICD-10) codes S72.0 (femoral neck fracture), S72.1 (pertrochanteric fracture), and S72.2 (subtrochanteric fracture). Fractures were included regardless of trauma mechanism because the administrative database does not reliably allow differentiation between low- and high-energy trauma. Furthermore, among individuals aged 50 years and older, hip fractures are typically related to low-energy trauma and are therefore considered fragility fractures according to international guidelines [29].

Individual-level longitudinal identifiers were not available in the publicly extracted SIH-SUS dataset, and repeated hospitalizations for the same individual could not be formally excluded. To reduce potential double counting due to rehospitalizations, we restricted the analysis to hospitalizations with ICD-10 codes for hip fracture (S72.0–S72.2) in combination with primary procedures representative of acute hip fracture management. Procedures considered indicative of index fracture treatment were selected based on clinical relevance and defined in consultation with a panel of orthopedists with extensive experience in the Brazilian public health system (Supplementary Table 1). This approach aimed to preferentially capture incident fracture events while minimizing inclusion of secondary procedures related to complications or revision surgeries.

Incidence rates were calculated per 100,000 population and stratified by age (5-year age intervals) and sex. A subgroup analysis focused on individuals aged 50 years and older.

### DXA utilization

DXA exams were identified using the official SUS procedure code 0204060028 (dual-energy X-ray absorptiometry), according to the Brazilian System of Management of the Table of Procedures, Medications, and Orthoses, Prosthetics and Special Materials (SIGTAP). This code is recorded in SIA-SUS for administrative reporting and reimbursement of outpatient procedures within the public healthcare system [30].

DXA utilization rates were stratified by age and sex, using 5-year age intervals. Subgroup analyses focused on individuals aged 50 years and older, as well as women aged 65 years and older and men aged 70 years and older, corresponding to internationally recommended screening thresholds.

The database includes the total number of DXA exams performed but does not allow identification of individual patients. Therefore, repeated examinations in the same individual may be included.

### DXA device availability

The annual number of DXA scanners from 2008 to 2022 was extracted from CNES. Devices were classified according to healthcare sector (public/SUS and private/non-SUS), and trends in distribution and growth were analyzed over time.

### Statistical analysis

The median age at hip fracture hospitalization and at DXA examination was calculated separately for men and women.

Odds ratios for hip fractures were calculated by comparing each age group with all younger individuals within the same sex (e.g., individuals aged 50–54 years were compared with those aged < 50 years).

Age-standardized hip fracture rates for individuals aged 50 years and older were calculated for each year of the study period using the World Health Organization (WHO) Standard Population (2000–2025) [31].

All analyses were performed using R version 4.3.0. The full analytical code is publicly available at: https://github.com/bc-teixeira/Osteoporosis.

## Results

### Hip fracture incidence

In this study (2008–2022), 583,205 hip fractures were registered in individuals aged 50 years and older covered by the SUS. The annual incidence rate of hip fractures in this age group was 134 per 100,000 women and 80 per 100,000 men. In 2022, the incidence rate was 155 per 100,000 women and 94 per 100,000 men aged 50 years and older; among women aged 65 years and older, the rate was 354 per 100,000, whereas in men aged 70 years and older, it was 255 per 100,000. The hip fracture rate per 100,000 individuals for each age group and sex in 2022 is detailed in Supplementary Table 2.

The median age of hip fracture cases in 2022, considering individuals aged 50 years and older, was 80 years (IQR = 72–86) for women and 74 years (IQR = 63–82) for men.

**Fig. 1:**
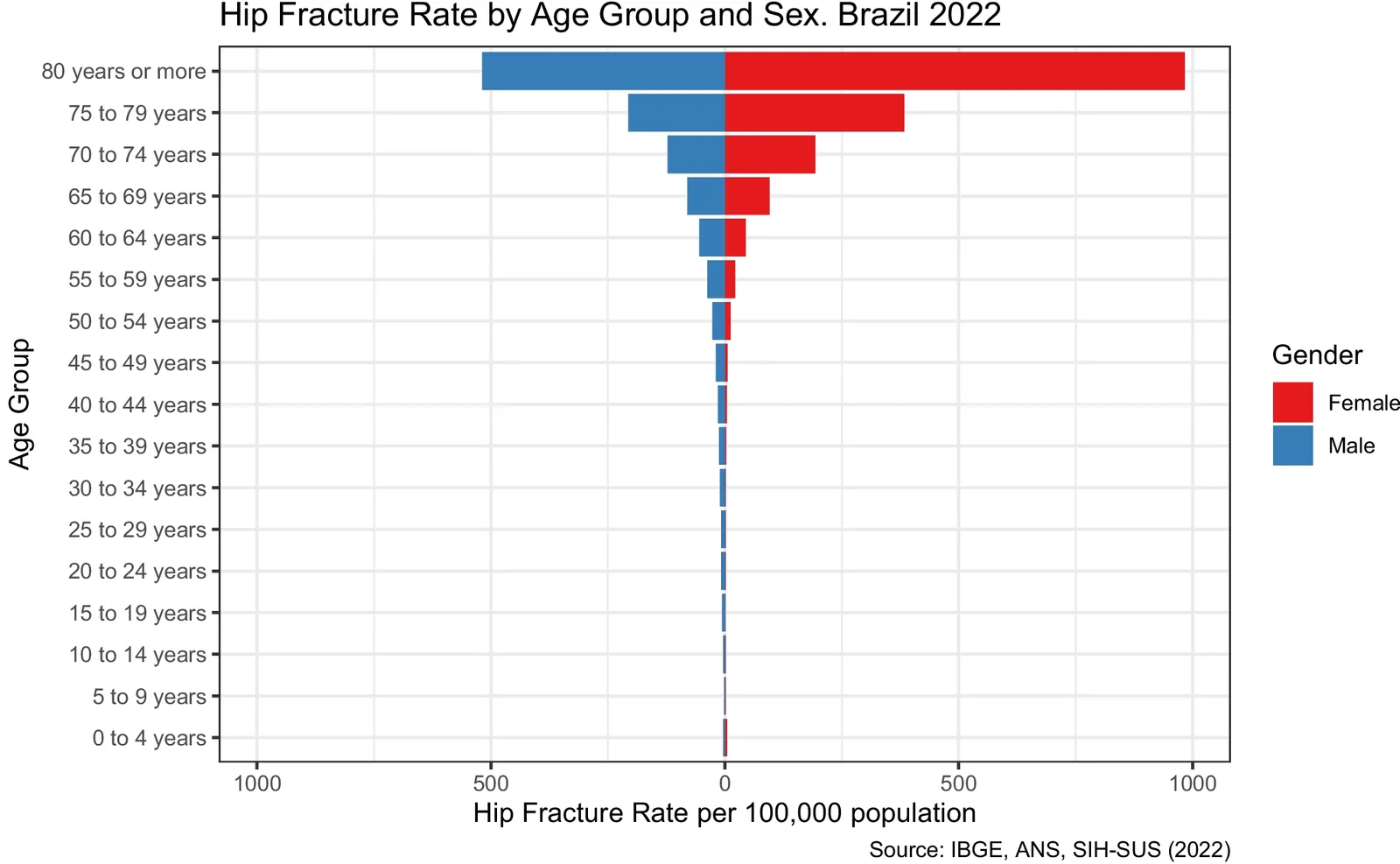
Hip fracture rate stratified by sex and age groups from 0 to over 80 years, per 100,000 population (2022)

Figure 1 shows the hip fracture rate by age group and sex in 2022. Hip fracture incidence in men begins to increase after age 45 and is slightly higher than in women up to age 64. From age 65 onwards, rates increase sharply in both sexes, with a more pronounced rise in women.

### Age-specific hip fracture risk

**Fig. 2:**
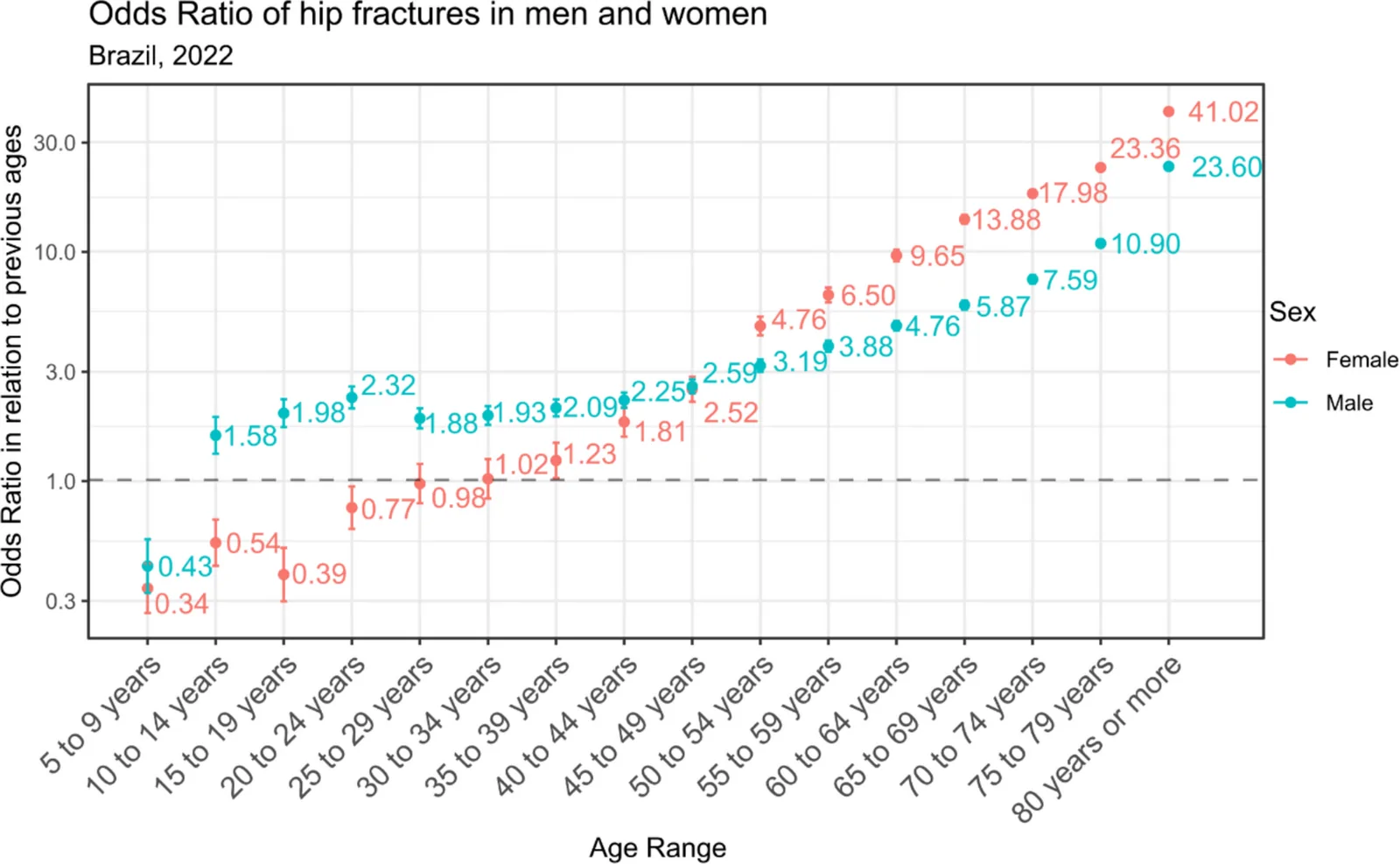
Odds ratios for hip fracture in men and women compared with those in all younger age groups (2022)

Each age group’s hip fracture risk was compared with the cumulative risk observed in all younger age groups, as shown in Fig. 2.

The risk of hip fracture in men increases progressively across age groups, with a more pronounced acceleration after age 50. Risk analysis revealed a 3.19-fold increase in hip fractures for men aged 50–54 years compared with all males under 50 years.

In women, the risk increases progressively across age groups, with a marked increase after 40 years. The odds of hip fracture in women aged 50–54 years are 4.76 times greater than those in all females under 50 years.

### Temporal trends in hip fracture incidence

Between 2008 and 2022, the absolute number of hip fractures among individuals aged 50 years and older increased substantially. The number of fractures increased by 28% between 2008 and 2015 and by 53% between 2015 and 2022. In 2022, a total of 55,643 hip fractures were recorded in this age group.

**Fig. 3:**
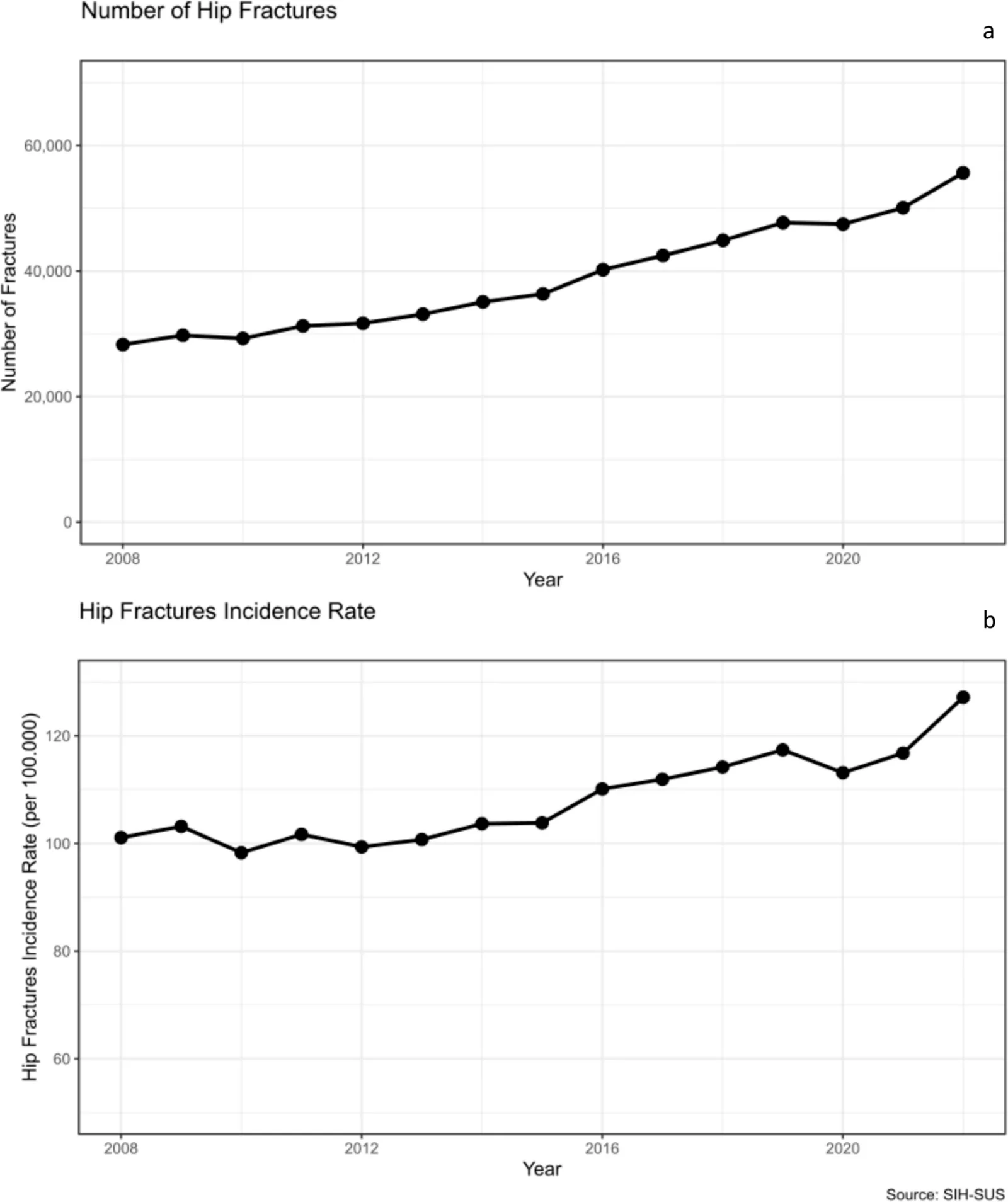
Hip fracture incidence in individuals aged 50 years and older in Brazil (2008–2022). The annual number of hip fractures recorded in the Brazilian public healthcare system (**a**) and the annual hip fracture incidence rate per 100,000 population (**b**)

Despite the increase in the absolute number of fractures, the hip fracture incidence rate per 100,000 population remained relatively stable from 2008 to 2015. From 2015 onward, the incidence rate increased progressively. A temporary decrease in hip fracture incidence was observed in 2020, followed by a recovery in 2021 and 2022 (Fig. 3a and 3b).

### Age-standardized hip fracture rates

The actual and standardized hip fracture rates were analyzed from 2008 to 2022 to assess the impact of population aging on the observed increase in fractures. Age-standardized rates were calculated for each year of the study period using the direct standardization method and the World Health Organization (WHO) Standard Population (2000–2025).

The hip fracture rate among individuals aged 50 and older increased from 101 per 100,000 in 2008 to 127 per 100,000 in 2022, corresponding to a 25.8% increase over the study period. After adjustment for the age distribution of the WHO Standard Population, the standardized rate in 2022 was 124.6 per 100,000.

The small difference between crude and standardized rates suggests that population aging contributed only modestly to the observed increase in hip fracture incidence during the study period (Supplementary Fig. 1 and Supplementary Table 3).

### DXA utilization

From 2008 to 2022, 6,318,743 DXA exams were performed in individuals aged 50 years or older through the SUS. Among women aged 50 years or older, the bone densitometry rate per 100,000 was 2092 exams, whereas among men in the same age group, it was significantly lower at 148 exams per 100,000.

In 2022, rates reached 2177 in women and 182 in men aged 50 years and older. Among individuals in the age groups recommended for osteoporosis screening—women aged 65 years or older and men aged 70 years or older—the DXA rates were 2840 and 385 exams per 100,000, respectively.

The DXA rate per 100,000 people for each age group and sex in 2022 is detailed in Supplementary Table 4. The median age at the time of the DXA exams in 2022, considering individuals aged 50 and older, was 64 years (IQR = 55–73) for women and 66 years (IQR = 54–75) for men.

The database reports the number of DXA examinations rather than individual patients; therefore, repeated examinations in the same individual may be included, potentially leading to an overestimation of rates.

### Temporal trends in DXA utilization

**Fig. 4:**
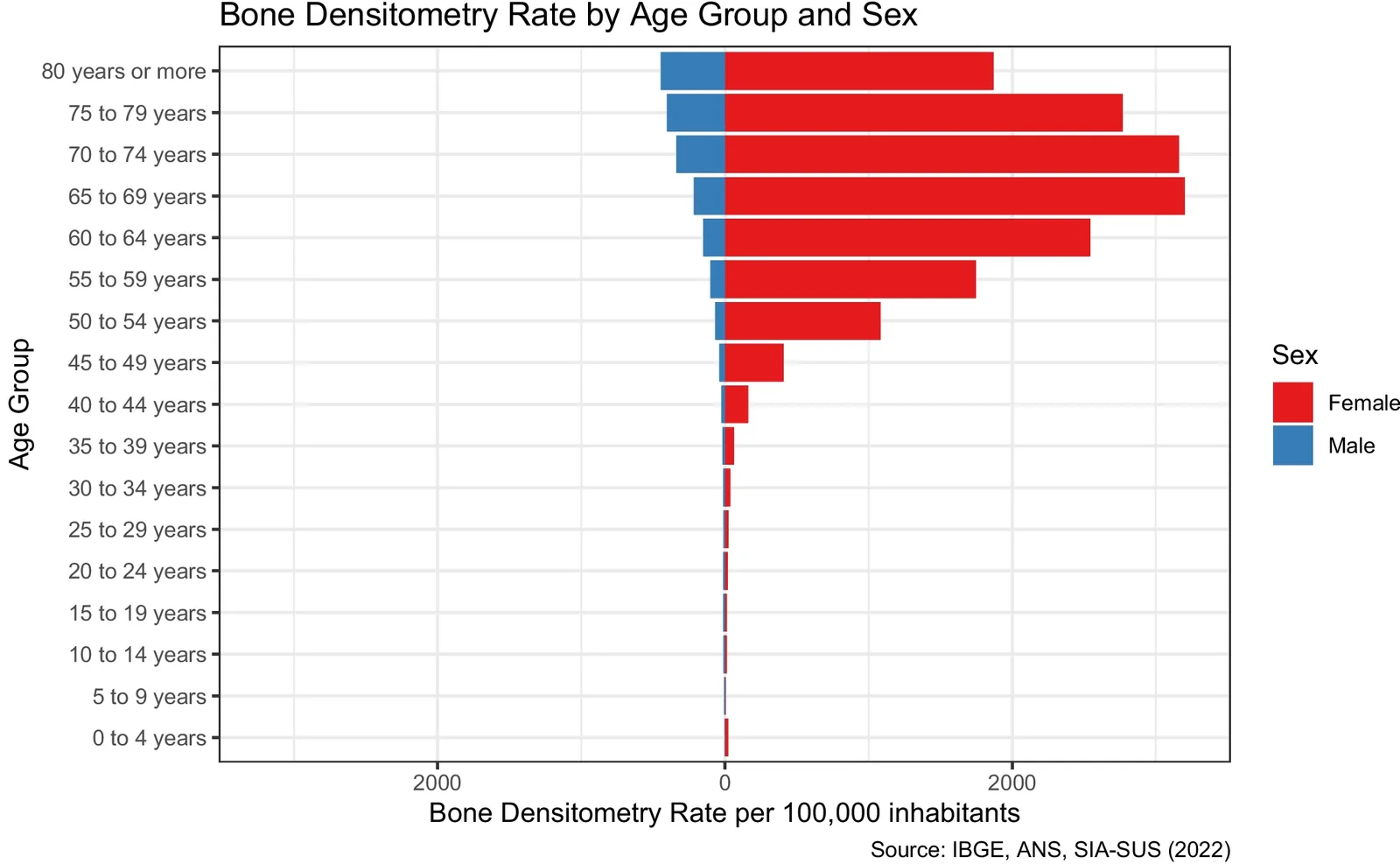
Bone densitometry rate stratified by sex and age groups from 0 to over 80, per 100,000 population (2022)

**Fig. 5:**
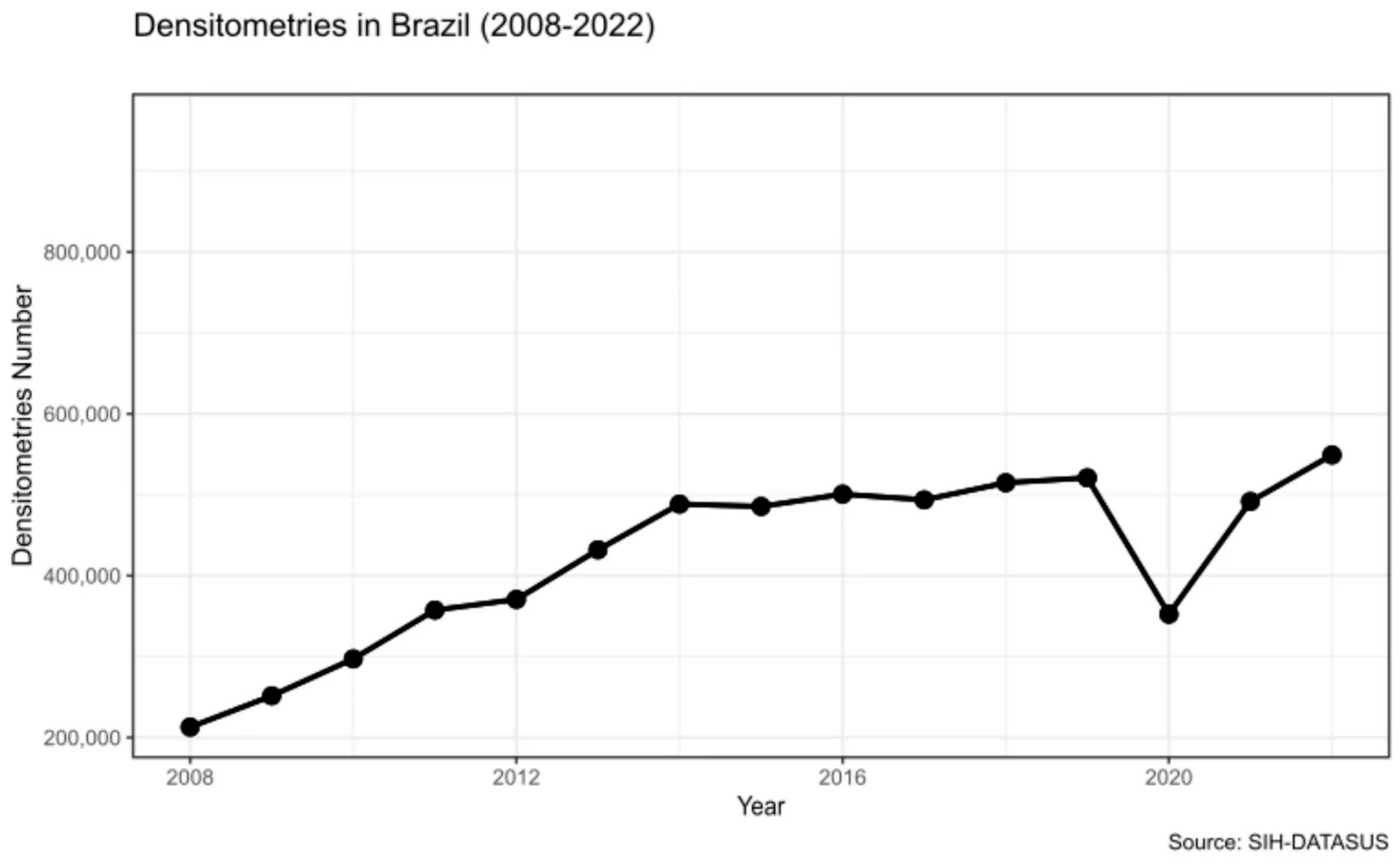
Trajectory of bone densitometry exams performed in Brazil from 2008 to 2022

The rate of DXA exams was consistently lower in men across all age groups (Fig. 4). The trajectory of the number of DXA exams in Brazil from 2008 to 2022 is shown in Fig. 5. Between 2008 and 2014, the total number of DXA exams increased by 129.5%, with an annual growth rate of 15%. From 2014 to 2022, the growth rate slowed to 12.4%, with an annual rate of 1.5%. In 2020, a 32% reduction in exams was observed compared with 2019. DXA utilization recovered in 2021 and surpassed pre-pandemic levels in 2022.

### Male–female disparities in fracture incidence and DXA utilization

**Fig. 6:**
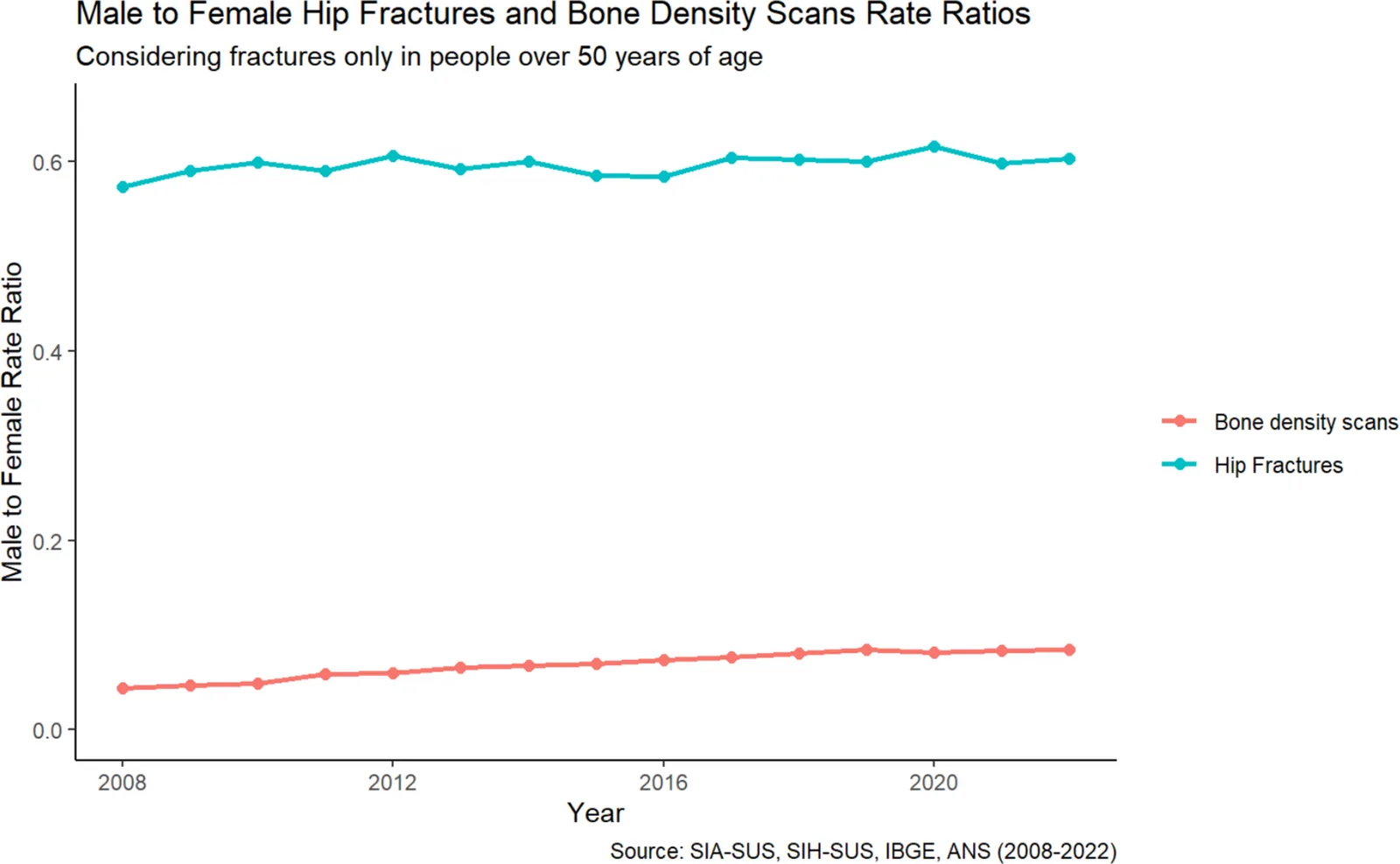
Ratio of male-to-female hip fracture rates (blue line) and bone densitometry scan rates ratio (red line) in individuals aged 50 years and older, 2008–2022

The representation of male-to-female hip fracture and bone densitometry scan rate ratios in individuals over 50 years of age revealed a significant discrepancy between the incidence of hip fractures and the rate of bone densitometry examinations in men (Fig. 6). The hip fracture rate ratio remained stable at approximately 0.6, meaning that for every 100 hip fractures in women, there were 60 in men. In contrast, the DXA scan rate ratio remained below 0.1, indicating that for every 100 DXA scans performed in women, 10 were conducted in men.

### DXA device availability

A substantial disparity in DXA device availability was observed between the SUS and the private healthcare sector from 2008 to 2022 (Supplementary Figs. 2 and 3). In 2022, the overall availability of DXA devices in Brazil was 12.2 per 1,000,000 inhabitants. When stratified by healthcare sector and adjusted for population coverage, the SUS had 6.2 devices per 1,000,000 individuals, whereas the private sector reached 27 devices per 1,000,000 individuals.

## Discussion

This study provides an updated analysis of hip fracture incidence by age and sex and DXA utilization in Brazil from 2008 to 2022, highlighting critical disparities between sexes and revealing a significant imbalance in the availability of DXA scanners between the public and private healthcare sectors. Our national estimates are consistent with previous Brazilian reports, including the Brazilian Osteoporosis Study (BRAVOS), which were used to recalibrate the Brazilian FRAX tool, as well as more recent analyses using administrative databases such as those of Sing et al. [22, 32]. However, our study extends these findings by incorporating more recent data through 2022 and by examining DXA utilization patterns and device availability across healthcare sectors.

An important finding was the imbalance between fracture burden and screening coverage in men. Despite men accounting for a substantial proportion of hip fractures among individuals aged 50 and older, their DXA screening rates remain disproportionately low, even within the age group recommended by international guidelines, which advise DXA screening for women aged 65 and older and men aged 70 and older [9, 10]. Although hip fracture incidence in women aged 65 + was only 1.4 times higher than that in men aged 70 +, our 2022 data showed that DXA screening was seven times more frequent in women. This corresponds to an approximately 3.5-fold difference in DXA utilization between men and women. The median age at which DXA exams were performed in men in 2022 was 66 years, which preceded the recommended screening age of 70 years. This may suggest that DXA exams in men are not routinely performed but are conducted in the context of other clinical conditions, with the overall number still far lower than in women.

Our results align with the internationally observed pattern of lower osteoporosis screening in men than in women [11, 12, 33–35]. Even following a hip fracture, men remain significantly underscreened and undertreated, with women being five times more likely to undergo DXA exams and nearly four times more likely to receive subsequent osteoporosis treatment [33].

This disparity in screening may partially explain the observed differences in hip fracture incidence between sexes. In our analysis, the male-to-female hip fracture rate ratio among individuals aged 50 years and older remained stable at approximately 0.60 throughout the study period. These findings align with the report by Sing et al., which identified Brazil, Thailand, and the UK as having the lowest overall hip fracture rates among 19 countries. However, Brazil had a higher male-to-female fracture ratio (0.60) than Thailand (0.46) and the UK (0.43), which may reflect the precariousness of diagnosing osteoporosis in men [22].

The higher male-to-female fracture ratio in Brazil is more likely related to insufficient osteoporosis screening and prevention efforts than to changes in fracture timing or improvements in male longevity. Women’s median age at hip fracture increased slightly from 79 to 80 years between 2008 and 2022, whereas men’s median age at hip fracture remained unchanged at 74 years. Although life expectancy increased modestly in Brazil during the study period, the unchanged median age at hip fracture among men suggests no corresponding delay in fracture onset. These observations are consistent with recent Danish data, which also reported a gradual increase in the mean age at femoral neck fracture among women (from 79.6 to 80.4 years), with no significant change in men (77 years), which was attributed to greater access to osteoporosis screening and more widespread treatment availability for women than for men [36].

Our findings indicate distinct patterns of hip fracture incidence between men and women according to age. Men presented higher fracture rates up to the age group of 60–64 years, after which rates increased more sharply in women. This pattern aligns with previous studies demonstrating that men typically fracture at younger ages [22, 33, 37, 38].

The temporal analysis of hip fracture trends further underscores the importance of addressing osteoporosis beyond demographic aging. Although aging contributes to higher absolute fracture numbers, our age-standardized incidence rates indicate that demographic shifts explain only a modest proportion of the observed increase. This suggests the involvement of additional factors, such as underdiagnosis of osteoporosis, inadequate treatment, or lifestyle shifts.

The stagnation in osteoporosis screening rates within the SUS since 2015, reflected in the decline in DXA exam growth to just 1.5% between 2014 and 2022, may partially account for the increasing fracture rates. A similar stagnation trend was observed in a US study, which reported that reductions in osteoporosis screening and treatment coincided with an increased burden of osteoporotic fractures [39]. Concerns over adverse effects associated with long-term bisphosphonate use may have further contributed to reduced treatment initiation and adherence, potentially influencing fracture trends [40].

The International Osteoporosis Foundation (IOF) recommends 10–11 DXA scanners per million inhabitants [2]. In 2022, Brazil met this target with 12.2 scanners per million when both the public and private sectors were considered. However, this overall figure masks a marked structural inequality in access. The public health system, which serves approximately 70% of the population, had only 6.2 scanners per million, well below the recommended level, whereas the private sector has 27 scanners per million. This disparity is not only a limitation in service provision, but also an important finding, as it highlights inequitable access to osteoporosis screening and fracture prevention according to healthcare coverage. Unequal geographic distribution of DXA scanners likely represents an additional barrier, particularly in underserved and rural areas [13].

The COVID-19 pandemic further complicated osteoporosis care, influencing DXA utilization and fracture incidence in Brazil. We documented a 32% decrease in DXA exams in 2020, possibly due to pandemic restrictions and reallocation of healthcare resources. Interestingly, hip fracture incidence temporarily decreased during the same period, potentially reflecting reduced mobility and fall risks amid social isolation, mirroring international findings [41, 42]. Gittoe et al. and Ramachandran et al. reported similar temporary reductions in fracture admissions early in the pandemic [41, 42]. However, subsequent increases in fracture incidence that we noted from 2021 to 2022 might reflect delayed consequences of interrupted osteoporosis care, underscoring the importance of sustained chronic disease management during healthcare crises.

Our findings are consistent with previous Brazilian estimates, including BRAVOS, and with more recent analyses using administrative databases [22, 24]. Differences across studies may be partly explained by methodological variations, particularly in population denominators and case definitions. For example, some studies excluded fractures associated with higher-energy trauma, which may not be reliably distinguishable from fragility fractures in older adults [23]. In our study, case identification was further refined by using both diagnostic codes and procedures representative of initial hip fracture management, which may improve specificity compared with approaches based on diagnosis codes alone [43].

In the multinational analysis by Sing et al., Brazil was among the countries with the lowest age-standardized hip fracture incidence rates, substantially lower than those reported in Northern Europe and parts of Asia [22]. Despite these relatively lower rates, demographic aging is expected to substantially increase the absolute number of hip fractures in many countries over the coming decades. These findings highlight the importance of strengthening fracture prevention strategies, particularly in settings where osteoporosis screening remains limited.

The sharp increase in hip fractures after age 50 in both men and women underscores the growing role of bone fragility, reinforcing well-established patterns in women while representing a paradigm shift for men. In our dataset, hip fracture risk in men increased progressively with age, with a significant acceleration after 50 years, reaching 3.19 times the baseline risk in the 50–54 age group.

Limitations of this study include the inability to determine whether DXA scans were performed before or after fracture, which prevents distinguishing between primary and secondary prevention. The mechanism of injury could not be determined, as administrative databases do not reliably distinguish between low- and high-energy trauma. Administrative databases also do not provide individual information on private insurance coverage at the time of hospitalization; therefore, some degree of misclassification in population estimates may occur. Because the publicly available SIH-SUS extracts do not include longitudinal patient identifiers, repeated hospitalizations for the same individual could not be formally excluded. We attempted to mitigate this limitation by restricting analyses to procedures representative of initial hip fracture management in combination with diagnostic codes; however, some residual repeated hospitalizations may persist. The data reflect total exam numbers without accounting for repeated tests, possibly inflating rates. Treatment rates were not assessed due to data unavailability. Postfracture mortality was not evaluated, although only in-hospital mortality is available in administrative databases. Comorbidities, underlying causes of osteoporosis, and potential underreporting or incomplete entries in the DATASUS system were also not assessed.

Our study’s main strengths are its nationwide coverage using large administrative databases, its long observation period, and its comprehensive assessment of hip fracture incidence, DXA utilization, and DXA device availability within the Brazilian healthcare system. Our findings contribute to the understanding of osteoporosis care and fracture prevention in Brazil by revealing long-term trends in hip fracture incidence and persistent gaps in DXA utilization across sexes and in scanner availability within the public healthcare system. By highlighting the limited availability of diagnostic tools in the SUS and the lower screening rates among men, this study adds evidence to discussions on equitable healthcare access and osteoporosis awareness.

This study was limited to hip fracture and DXA utilization data from the Brazilian public healthcare system (SUS), which was the primary focus of our analysis. DXA device availability, however, included both public and private healthcare sectors. While structural limitations such as scanner availability may partially explain the observed gender disparity, they do not fully account for it. Future research should investigate whether this gap persists in the private sector, where access is broader, to help clarify the extent to which sociocultural and clinical factors contribute to the underscreening of men for osteoporosis.

## Conclusion

This study provides a nationwide overview of hip fracture incidence and DXA utilization trends in Brazil between 2008 and 2022, with a 25.8% increase in the crude hip fracture rate over the study period. In 2022, the incidence of hip fracture was 94 per 100,000 in men and 155 per 100,000 in women aged ≥ 50, with median fracture ages of 74 and 80 years, respectively. Although the hip fracture incidence ratio between men and women was 2:3 (0.6), the number of DXA scans performed between sexes was 1:10 (0.1). In addition, marked disparities in device availability between the public and private sectors were demonstrated. These findings highlight structural limitations within the public healthcare system and suggest persistent underdiagnosis of osteoporosis, with an even more pronounced gap in men, a pattern also observed in high-income countries. Addressing this disparity will require coordinated efforts, including prioritization of male osteoporosis in screening strategies and further research into barriers and potential solutions across healthcare systems.

## Supplementary Information

Below is the link to the electronic supplementary material.

Supplementary Material 1 (DOCX 319 KB)

## Data Availability

The datasets analyzed in this study are publicly available from the Brazilian Ministry of Health’s DATASUS platform, including SIH-SUS, SIA-SUS, CNES, and population data, and from the National Supplementary Health Agency (ANS). The analytical code is publicly available at https://github.com/bc-teixeira/Osteoporosis.
