# Supplementary material for: Sex-specific trends in hip fracture incidence and disparities in DXA utilization in Brazil: a nationwide ecological analysis
Source: Arch Osteoporos. 2026 Aug 26;21(1):128. doi: 10.1007/s11657-026-01734-5 (PMC13518430; doi:10.1007/s11657-026-01734-5)
Supplement: Supplementary file 1 [file 11657_2026_1734_MOESM1_ESM.docx]

**Sex-specific trends in hip fracture incidence and disparities in DXA utilization in Brazil: a nationwide ecological analysis**

Journal name: Archives of Osteoporosis

JANUÁRIO, I. - ISABELA JANUÁRIO– dra.isabelajanuario@gmail.com

LAZARETTI-CASTRO M. - MARISE LAZARETTI-CASTRO –lazaretti.castro@unifesp.br

ALBERGARIA, B. H. - BEN‑HUR ALBERGARIA - - benhur.gaz@terra.com.br

TEIXEIRA, B. C. - BRUNO CASAES TEIXEIRA - - bcasteix@gmail.com

Corresponding author: BEN‑HUR ALBERGARIA, Federal University of Espírito Santo - benhur.gaz@terra.com.br

**Supplementary informations**

To validate and refine the identification of hip fractures, broader ICD-10 codes for fractures (e.g., S02, S12, S22, S32, S42, S52, S62, S82, S92) were initially included. These codes were used to differentiate hip fractures from other fracture types and ensure methodological rigor.

The regex pattern ^S.{1}2.{0,1}$ was applied exclusively to ICD-10 codes in the datasets to identify hospitalizations related to fractures. Procedural codes were selected separately using predefined lists of codes corresponding to surgical and non-surgical treatments associated with fractures.

An initial selection of codes was performed to include all primary procedures related to fracture care to avoid counting re-hospitalizations. This broad selection encompassed various types of care associated with fractures. Subsequently, a second filter was applied to isolate procedures related explicitly to hip fractures, excluding those related to secondary care or complications. This classification was refined with input from a panel of orthopedists with extensive experience in SUS practices.

**Supplementary Table 1.**Targeted procedures focusing on the care of the hip fracture

| Procedure Code | Procedure Description | Procedure Group | Procedure Subgroup | Procedure Form |
| --- | --- | --- | --- | --- |
| 408050632 | SURGICAL TREATMENT OF INTERTROCHANTERIC FRACTURE | Surgical procedures | Musculoskeletal system surgery | Lower limbs |
| 408040050 | PARTIAL HIP ARTHROPLASTY | Surgical procedures | Musculoskeletal system surgery | Pelvis |
| 408050489 | SURGICAL TREATMENT OF PROXIMAL FEMORAL FRACTURE / PHYSIS LESION (NECK) (FIXATION) | Surgical procedures | Musculoskeletal system surgery | Lower limbs |
| 408040092 | PRIMARY TOTAL HIP ARTHROPLASTY (NON-CEMENTED / HYBRID) | Surgical procedures | Musculoskeletal system surgery | Pelvis |
| 408040084 | PRIMARY TOTAL HIP ARTHROPLASTY (CEMENTED) | Surgical procedures | Musculoskeletal system surgery | Pelvis |
| 408050616 | SURGICAL TREATMENT OF SUBTROCHANTERIC FRACTURE | Surgical procedures | Musculoskeletal system surgery | Lower limbs |
| 408050519 | SURGICAL TREATMENT OF FEMORAL SHAFT FRACTURE | Surgical procedures | Musculoskeletal system surgery | Lower limbs |
| 408050233 | CLOSED REDUCTION OF FEMORAL SHAFT FRACTURE / PROXIMAL PHYSIS INJURY | Surgical procedures | Musculoskeletal system surgery | Lower limbs |
| 408040068 | TOTAL HIP ARTHROPLASTY REVISION | Surgical procedures | Musculoskeletal system surgery | Pelvis |
| 408040262 | SURGICAL TREATMENT OF HIP FRACTURE / DISLOCATION / FRACTURE-DISLOCATION / SYMPHYSEAL DISRUPTION | Surgical procedures | Musculoskeletal system surgery | Pelvis |
| 408050586 | SURGICAL TREATMENT OF FRACTURE OF THE FEMORAL CONDYLE / CONDYLES | Surgical procedures | Musculoskeletal system surgery | Lower limbs |
| 408040041 | NON-CONVENTIONAL HIP ARTHROPLASTY | Surgical procedures | Musculoskeletal system surgery | Pelvis |

**Supplementary Table 2**. Hip fracture rate by age group and sex in the year 2022.

**Supplementary Figure 1.** The graph illustrates the trends in actual and standardized hip fracture rates in Brazil from 2008 to 2022 for individuals aged 50 and older. The red line represents the actual incidence rate, calculated based on the number of hip fractures per 100,000 individuals covered by the public healthcare system (SUS). The blue line represents the standardized rate, adjusted to the WHO Standard Population (2000–2025) to account for demographic changes over time. Both rates show a gradual increase over the study period, with the actual and standardized rates remaining closely aligned. This indicates that population aging had a limited impact on the increase in hip fracture incidence, as the difference between the two curves remains minimal.

**Supplementary Table 3**. Standardised and actual fracture rates, 2008-2022.

**Supplementary** **Table 4**. Bone densitometry rate by age group and sex in the year 2022

**Supplementary figure 2.** Annual Number of Densitometry Devices in SUS and Non-SUS from 2008 to 2022.

**Supplementary figure 3**. Density of Densitometry Devices per 100,000 People Aged 50+ in SUS and Non-SUS from 2008 to 2022.
